# Working memory related functional connectivity in adult ADHD and its amenability to training: A randomized controlled trial

**DOI:** 10.1016/j.nicl.2024.103696

**Published:** 2024-11-02

**Authors:** Tuija Tolonen, Sami Leppämäki, Timo Roine, Kimmo Alho, Pekka Tani, Anniina Koski, Matti Laine, Juha Salmi

**Affiliations:** a Department of Psychology, University of Helsinki, Helsinki, Finland; b Department of Neuroscience and Biomedical Engineering, Aalto University, Espoo, Finland; c AMI Centre, Aalto Neuroimaging, Aalto University, Espoo, Finland; d Terveystalo Healthcare, Helsinki, Finland; e Department of Psychiatry, Helsinki University Hospital, Helsinki, Finland; f Department of Psychology, Åbo Akademi University, Turku, Finland; g Unit of Psychology, Faculty of Education and Psychology, University of Oulu, Oulu, Finland

**Keywords:** Adult ADHD, Working memory, fMRI, Network-based statistic, Connectivity, Cognitive training

## Abstract

**Background:**

Working memory (WM) deficits are among the most prominent cognitive impairments in attention deficit hyperactivity disorder (ADHD). While functional connectivity is a prevailing approach in brain imaging of ADHD, alterations in WM-related functional brain networks and their malleability by cognitive training are not well known. We examined whole-brain functional connectivity differences between adults with and without ADHD during *n*-back WM tasks and rest at pretest, as well as the effects of WM training on functional and structural brain connectivity in the ADHD group.

**Methods:**

Forty-two adults with ADHD and 36 neurotypical controls performed visuospatial and verbal *n*-back tasks during functional magnetic resonance imaging (fMRI). In addition, seven-minute resting state fMRI data and diffusion-weighted MR images were collected from all participants. The adults with ADHD continued into a 5-week randomized controlled WM training trial (experimental group training on a dual *n*-back task, *n* = 21; active control group training on Bejeweled II video game, *n* = 21), followed by a posttraining MRI. Brain connectivity was examined with Network-Based Statistic.

**Results:**

At the pretest, adults with ADHD had decreased functional connectivity compared with the neurotypical controls during both *n*-back tasks in networks encompassing fronto-parietal, temporal, occipital, cerebellar, and subcortical brain regions. Furthermore, WM-related connectivity in widespread networks was associated with performance accuracy in a continuous performance test. Regarding resting state connectivity, no group differences or associations with task performance were observed. WM training did not modulate functional or structural connectivity compared with the active controls.

**Conclusion:**

Our results indicate large-scale abnormalities in functional brain networks underlying deficits in verbal and visuospatial WM commonly faced in ADHD. Training-induced plasticity in these networks may be limited.

## Highlights

• Adults with ADHD had decreased functional connectivity during working memory tasks.
• Aberrant networks encompassed areas associated with working memory and ADHD.
• No differences in functional connectivity during resting state.
• Working memory training did not affect functional or structural connectivity.

## 1 Introduction

Attention deficit hyperactivity disorder (ADHD) is manifested as difficulties to maintain attention, impulsive behavior, and hyperactivity (American Psychiatric Association 2013). No reliable objective behavioral markers for ADHD have been found, but deficits in working memory (WM) are suggested to be a key factor contributing to the core symptoms (Willcutt et al., 2005). WM enables mental maintenance and manipulation of information and supports many complex behaviors needed in everyday life (D’Esposito and Postle, 2015). Poor WM is associated with severe attention problems (Alloway et al., 2009, Kofler et al., 2010, Tillman et al., 2011) and has widespread effects, for instance, on academic performance (Alloway and Alloway, 2010; Rogers et al., 2011) and organizational skills (Kofler et al., 2018). Prevailing etiological theories suggest that ADHD symptoms emerge from complex network-level aberrancies in brain structure and function (see, e.g., Faraone et al., 2015, Sutcubasi et al., 2020), but whole-brain connectomics related to WM performance are still poorly understood. Another key issue to address in cognitive neuroscience research is the malleability of abnormal networks to behavioral interventions, as standard psychopharmaceutical treatments are sometimes ineffective and can have unpleasant adverse effects (Banaschewski et al., 2004, Brinkman et al., 2018).

Adults with ADHD have difficulties with both verbal and visuospatial WM (Alderson et al., 2013). One of the most common WM paradigms used in brain research (Wang et al., 2019, Yaple and Arsalidou, 2018, Yaple et al., 2019) is the *n*-back task, which requires continuous updating of WM content by determining whether or not a current item in a stimulus stream matches the one presented *n* items ago (Kirchner, 1958). For example, in a visuospatial 2-back task, a participant’s task is to determine whether a current stimulus occurs in the same location as the stimulus presented just before the preceding one. *n*-back tasks activate the prefrontal and parietal cortices, insula, cerebellum, thalamus, and cingulate cortex (Wang et al., 2019, Yaple and Arsalidou, 2018, Yaple et al., 2019), overlapping the brain areas of a more general WM network (Rottschy et al., 2012). Activation patterns show considerable similarities across different WM task types (Rottschy et al., 2012, Wang et al., 2019). However, the left inferior frontal gyrus has shown enhanced activity during verbal vs. nonverbal WM and the supplementary motor area during nonverbal vs. verbal WM (Rottschy et al., 2012). Moreover, visuospatial WM tasks have shown increased activity in the anterior insula and cerebellar tonsil compared with an object identification WM task (Wang et al., 2019). The brain areas engaged in WM tasks, especially the prefrontal cortex and insula, are usually less active during WM performance in individuals with ADHD than in neurotypical (NT) participants (Cortese et al., 2012, McCarthy et al., 2014; Tamon et al., 2024). In the cerebellum and posterior cingulate cortex, enhanced activations have also been observed in individuals with ADHD (Tamon et al., 2024). Regional activity changes in many WM hubs were also found with the current adult ADHD sample in a previous study (Salmi et al., 2020). WM involves multiple cognitive component processes that operate with spatial and verbal WM slave systems, relying on synchronized activation between several different brain networks (D’Esposito and Postle 2015). Therefore, examining network-level brain systems can provide important information about the mechanisms of WM as well as related dysfunctions.

Only a few studies have investigated WM-related network-level functional connectivity in ADHD vs. NT individuals (Bédard et al., 2014, Kowalczyk et al., 2023, Massat et al., 2012, Wolf et al., 2009, Wu et al., 2017). Functional connectivity refers to synchronized activation patterns between brain areas, determined as, for instance, temporal correlations between blood-oxygen level-dependent (BOLD) signals measured in different brain areas with functional magnetic resonance imaging (fMRI) (Rogers et al., 2007). The results of WM-related functional connectivity studies in individuals with ADHD have been somewhat inconsistent, showing both increased (Bédard et al., 2014, Kowalczyk et al., 2023, Massat et al., 2012, Wolf et al., 2009, Wu et al., 2017) and decreased (Bédard et al., 2014, Wolf et al., 2009, Wu et al., 2017) connectivity, compared with NT individuals, in partially overlapping networks. Hyperconnected networks have included the prefrontal, temporal, parietal, occipital, insular, and cingulate cortices, as well as the cerebellum, striatum, and globus pallidus (Bédard et al., 2014, Kowalczyk et al., 2023, Massat et al., 2012, Wolf et al., 2009, Wu et al., 2017); reduced connectivity has encompassed the frontal, parietal, insular, and cingulate cortices, and the cerebellum (Bédard et al., 2014, Wolf et al., 2009, Wu et al., 2017). Moreover, all but one (Wolf et al., 2009) of the studies on functional connectivity changes in ADHD were conducted in children. Given that both functional connectivity patterns (Grayson and Fair, 2017) and ADHD symptoms evolve during childhood (Franke et al., 2018), it is difficult to make reliable inferences from child studies on WM-related functional connectivity in adults with ADHD. In the only study on adults, Wolf and colleagues (2009) found reduced connectivity in ADHD in fronto-parieto-cerebellar areas and the anterior cingulate cortex, and hyperconnectivity in many frontal areas and the dorsal cingulate cortex. Another limitation in previous studies is that most of them have investigated the functional connectivity of predefined areas or networks, possibly exaggerating their significance or excluding other relevant areas from the analysis. Other approaches, such as Network-Based Statistic (NBS; Zalesky et al., 2010), a data-driven clustering method used to explore the connectivity architecture of whole-brain networks with minimal a priori assumptions, might provide complementary information on the complex functional connectivity changes in neurodevelopmental disorders such as ADHD.

Due to the important role of WM deficits in the cognitive problems related to ADHD, WM training was seen as a potential candidate for ADHD rehabilitation already almost two decades ago (Klingberg et al., 2005). Since then, the behavioral effects of cognitive training on individuals with ADHD have been extensively studied. These studies have shown cognitive training related moderate improvements in untrained WM tasks, and some studies have even found evidence of transfer effects on inhibition control and attention which are some of the core ADHD symptoms hampering everyday life (Cortese et al., 2015, Pauli-Pott et al., 2021, Robledo-Castro et al., 2023, Westwood et al., 2023, Zou et al., 2024). It is less clear whether cognitive training is capable of remediating aberrant brain functioning in individuals with ADHD. A few cognitive training studies on children and adolescents with ADHD have reported volumetric increases from pretest to posttest in frontal and cerebellar gray matter (Hoekzema et al., 2011) and regional brain activity increases in frontal, parietal and temporal lobe regions and in the cerebellum (Hoekzema et al., 2010, Stevens et al., 2016) and decreases in the insula and subcortical structures (de Oliveira Rosa et al., 2020). Our previous results from the present randomized controlled trial showed that WM training restored regional activation in the fronto-parietal areas, precuneus, and supplementary motor area in adults with ADHD (Salmi et al., 2020). More specifically, we demonstrated that the training effects were different for the criterion task and for its untrained variant: increased activity was observed for the training task, and decreased activity for the near-transfer task (Salmi et al., 2020). This may partly explain the mixed results found in previous studies employing either a criterion task or a near-transfer task only. Besides regional effects, some studies with neurotypical adults have investigated how cognitive training affects structural or functional connectivity (for reviews, see Brooks et al., 2020, Constantinidis and Klingberg, 2016). To highlight a few, studies using the NBS method have revealed training-related increases in structural and functional connectivity in networks encompassing frontal, temporal, and parietal areas (Román et al., 2017, Zuber et al., 2021). However, the network-level neural modulation effects of WM training in individuals with ADHD have not yet been studied.

In the present study, we investigated differences in WM-related functional connectivity between adults with and without ADHD, as well as the malleability of these differences in response to WM training. WM was investigated within two of its core subsystems, visuospatial and verbal, to get a comprehensive view of connectivity patterns related to different WM components (Baddeley and Hitch, 1974). As mentioned above, brain activations related to the two components are somewhat dissimilar (Rottschy et al., 2012, Wang et al., 2019), but such differences have not previously been addressed in ADHD. Furthermore, WM training effects were anticipated to transfer mostly to tasks that are similar to the trained one (see e.g. Soveri et al., 2017a), and therefore, the two *n*-back variants of the trained dual *n*-back task (see below) were selected as outcome measures during fMRI. Additionally, we examined brain connectivity during resting state to further explore whether possible group differences during WM performance are explained by intrinsic brain connectivity. Functional connectivity was analyzed with the NBS method in two ways, as group-level differences between adults with ADHD and NT controls, and as correlations between functional connectivity, ADHD symptoms, and continuous performance test (CPT) scores regardless of the participant’s diagnostic status. CPT is the most widely studied objective behavioral marker of ADHD (Gualtieri and Johnson, 2005), and related behavioral indices that are correlated with symptoms (Albrecht et al., 2015; however, see Arrondo et al., 2024) were hence expected to provide complementary information about aberrant functional connectivity patterns. We expected to find widespread hypoconnectivity in adults with ADHD compared with the NT controls, based on our previous findings of ADHD-related reduced structural connectivity within the same sample (Tolonen et al., 2023). We also hypothesized that higher error rates in the CPT would be associated with reduced functional connectivity, also based on our previous findings of a corresponding association with structural connectivity (Tolonen et al., 2023).

To examine the effects of WM training, the participants with ADHD took part in a randomized controlled WM training trial, divided into an experimental group, training a visuospatial-verbal dual *n*-back task with a constantly adapting *n*-back level in response to the participant’s performance, and an active control group practicing a video game which required attention but not WM. We have previously reported training-induced changes in task performance and ADHD symptoms in the present sample (Salmi et al., 2020). Briefly, the experimental group with ADHD improved more than the active controls with ADHD in the trained dual *n*-back task and in an untrained spatial WM task, and in a subset of the experimental group (who improved less in the trained task) ADHD symptoms reduced more compared with the active controls. In the current study, training effects on brain connectivity were analyzed by examining the interaction between group (*n*-back training group vs. active control group) and session (pre- vs. posttraining MRI). In addition to functional connectivity, WM training effects on structural connectivity were also examined, building on our previous study of abnormal structural connectivity in adults with ADHD (Tolonen et al., 2023). We hypothesized that WM training would lead to increased functional connectivity. This was based on our hypothesis of weaker connectivity in ADHD adults than NT controls before training and the training-related restoration of regional brain activity previously reported in the same sample (Salmi et al., 2020), as well as earlier studies showing mainly increases in functional connectivity following WM training in NT individuals (Jolles et al., 2013, Sánchez-Pérez et al., 2019, Takeuchi et al., 2013, Thompson et al., 2016, Zuber et al., 2021). Although the evidence of changes in structural connectivity resulting from WM training is a bit more limited (Metzler-Baddeley et al., 2017, Román et al., 2017, Salminen et al., 2016, Takeuchi et al., 2010), the existing evidence for experience-induced neuronal plasticity (Sampaio-Baptista and Johansen-Berg, 2017) and its potential implications prompted us to conduct pre-post comparisons also for the networks extracted from the diffusion-weighted MR images.

## 2 Materials and methods

### 2.1 Participants

**Fig. 1:**
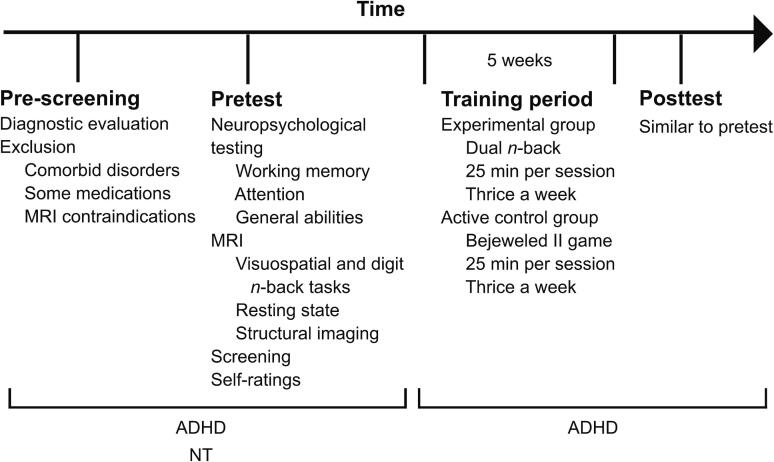
Schematic overview of the study stages. The study consisted of three parts: a pretest neuropsychological assessment and imaging session, a training period, and a posttest neuropsychological assessment and imaging session. Pre-screening was conducted before the pretest. The NT controls participated only in the pretest, while the participants with ADHD continued to the training period and the posttest. Image adapted from Salmi et al. (2020), published under CC BY 4.0 license.

Forty-two adults with ADHD and 36 NT controls participated in the study (see Supplementary Table 1 in Supplementary Results). The participants with ADHD were pre-screened and recruited by psychiatrists at the Neuropsychiatry outpatient clinic of the Helsinki University Hospital and at private clinics (Diacor Healthcare Services, Helsinki, Finland, and ProNeuron, Espoo, Finland). Comorbid disorders were excluded as part of a regular clinical assessment with Interview for DSM-IV Axis I Disorders (SCID-I) and the Mini-International Neuropsychiatric Interview (M.I.N.I.). Exclusion criteria included any other severe psychiatric or neurological disorders apart from ADHD, including head trauma requiring treatment, substance abuse, or other addictions. The NT participants were recruited via email lists at universities, polytechnics, adult high schools, and vocational schools, and via the authors’ personal contacts. All participants were native Finnish speakers, had normal or corrected-to-normal vision, had sufficient hearing, and met the MRI eligibility criteria. The NT adults who participated only in the pretest (see Fig. 1 for an overview of the study stages) were compensated by 60 €, and the ADHD participants continuing to the training period and the posttest by 240 €.

ADHD was diagnosed according to the Diagnostic and Statistical Manual of Mental Disorders, Fourth Edition (DSM-IV) and the participants’ current status was confirmed with the Conners’ Adult ADHD Diagnostic Interview for DSM-IV (Epstein et al., 2001). All participants with ADHD met the criteria for both inattention and hyperactivity or only for inattention. Four participants had migraine, two had hypothyroidism, and two had experienced mild epilepsy symptoms in childhood but had not needed treatment since that time. Four participants had a prescription for migraine medicine, one for mild depression (selective serotonin re-uptake inhibitor), and two for hypothyroidism. Thirty-three participants with ADHD were using stimulants. Participants were requested to have a 24-h wash-out period from psychostimulants before the pre- and posttests including the fMRI session.

### 2.2 Self-ratings and general abilities

ADHD symptoms were self-assessed with the Adult ADHD Self-Report Scale (ASRS) version 1.1 (Kessler et al., 2005), mood with the Depression Scale (DEPS; Salokangas et al., 1995), and alcohol consumption with the Alcohol Use Disorders Identification Test – Consumption (Bush et al., 1998). General cognitive abilities were assessed with the Matrix reasoning and Vocabulary tests of the Wechsler Adult Intelligence Scale (WAIS-III; Wechsler 2005).

### 2.3 fMRI conditions

#### 2.3.1 Resting state

During the resting state condition, participants were instructed to fixate their gaze to a white cross presented in the center of a gray screen. Otherwise, there was no task for the participants during the acquisition. The resting state run lasted for approximately seven minutes.

#### 2.3.2 Single *n*-back tasks

Two variants of single *n*-back tasks were presented during the fMRI session: a version with visuospatial material (white squares presented in eight locations of a 3 x 3 matrix where the fixation point occupied the center) and another with visually presented digits (from 1 to 9 presented at the center of the screen). In both task variants, the blocks consisted of four different *n*-back levels (0-, 1-, 2-, and 3-back, one level in one block) presented in a counterbalanced order (0-back, 1-back, 2-back, 3-back, 3-back, 2-back, 1-back, 0-back, etc.). All blocks began with an instruction of the *n*-back level. Each location/digit was shown on the screen for 1500 ms with an interstimulus interval (ISI) of 450 ms. Two response buttons, match and nonmatch, were used at load levels higher than 0-back. In the 0-back task, the participants pressed the nonmatch button for each stimulus. Each block consisted of 10 trials, of which randomly two on average were match trials (targets). Each task level was presented five times, and the duration of the task was approximately 13 min for each task variant.

### 2.4 Offline cognitive tasks

#### 2.4.1 Dual *n*-back task

Dual *n*-back task was conducted in the pretest (outside of the scanner) and was used as the training task for the experimental group. During the task, visuospatial material (same as in the visuospatial single *n*-back task; see Section 2.3.2) was presented simultaneously with phonological material (spoken Finnish syllables /dy/, /ki/, /le/, /næ/, /pø/, /ro/, /su/, or /ta/). The squares and the syllables were presented concurrently, with 500 ms stimulus presentation and 2,500 ms ISI. The participants were instructed to indicate whether the latest stimulus, either a square location, a syllable, or both, matched the one *n* trials back. They had to press the left key if the square location matched the location *n* trials back, the right key if the syllable matched the syllable *n* trials back, and both keys if both matched the items *n* trials back. The probabilities of matches and nonmatches were equal in both tasks. Total task duration was approximately 25 min, with 20 blocks of sequences, each containing 20 stimuli pairs. Task difficulty was continuously adjusted based on the participant’s performance. The level of *n* was between one and nine in each block. The program increased *n* by 1 in the following block when 90 % accuracy was reached and decreased by 1 if the number of correct answers fell below 75 % for either stimulus type. The starting level of each session was 2-back. A result screen showing the highest level of *n* reached during the session and the number of blocks completed for each *n* level was displayed at the end of the session.

#### 2.4.2 Continuous performance test

Continuous performance test (CPT) (Rosvold et al., 1956) was performed outside of the scanner. We used the implementation of the Conners Continuous Performance Test 2 (Conners and Staff, 2000) available in the Psychology Experiment Building Language (PEBL) toolbox (Mueller and Piper, 2014; RRID:SCR_014794). In this test, the participants were presented with a series of letters and instructed to press the space bar for each letter apart from the letter X, which occurred in 9.7 % of the trials. There were 360 trials (letters) with fixed alternating intervals (1000 ms, 2000 ms, and 4000 ms), and the duration of the task was approximately 14 min. We used two scores in this study: omission errors (failure to respond to a target letter) and commission errors (responding to a nontarget-letter X).

### 2.5 MRI acquisition

MRI data were collected at the Advanced Magnetic Imaging Centre (Aalto University) using a Siemens MAGNETOM Skyra 3 T scanner (Siemens Healthcare, Erlangen, Germany) mounted with a 30-channel head coil. Functional runs were acquired using gradient-echo echo planar imaging with the following parameters: repetition time (TR) 1.9 s, voxel matrix 64 × 64, slice thickness 3.0 mm, and in-plane resolution 3.1 mm × 3.1 mm. The two single *n*-back tasks were conducted in different runs, each including 408 volumes. The resting state run included 225 volumes. In addition to fMRI, an anatomical scan with a T1-weighted (T1w) MPRAGE sequence was obtained for registration purposes with the following parameters: TR 2.5 s, voxel matrix 256 × 256, slice thickness 1 mm, and in-plane resolution 1 mm × 1 mm.

The tasks presented during the functional runs were projected with a 3-micromirror data projector (Christie X3, Christie Digital Systems, Mönchengladbach, Germany) on a semitransparent screen located behind the participants’ head. The distance to the screen was approximately 34 cm via a mirror located above the eyes of the participant with a binocular field of view 24 cm.

### 2.6 Preprocessing of the imaging data

Results included in this paper come from preprocessing performed using fMRIPrep 22.1.0 (Esteban et al., 2019; RRID:SCR_016216), which is based on Nipype 1.8.5 (Gorgolewski et al., 2011; RRID:SCR_002502). Many internal operations of fMRIPrep use Nilearn 0.9.1 (Abraham et al., 2014; RRID:SCR_001362). The boilerplate text in this section (except for Section 2.6.3 Denoising) was automatically generated by fMRIPrep with the express intention that users should copy and paste this text into their manuscripts unchanged. It is released under the CC0 license.

#### 2.6.1 Anatomical data preprocessing

The T1w image was corrected for intensity non-uniformity (INU) with N4BiasFieldCorrection (Tustison et al., 2010), distributed with ANTs 2.3.3 (Avants et al., 2008; RRID:SCR_004757), and used as T1w reference throughout the workflow. The T1w reference was then skull-stripped with a Nipype implementation of the antsBrainExtraction.sh workflow (from ANTs), using OASIS30ANTs as target template. Brain tissue segmentation of cerebrospinal fluid (CSF), white-matter (WM) and gray-matter (GM) was performed on the brain-extracted T1w using fast (Zhang et al., 2001; FSL 6.0.5.1:57b01774, RRID:SCR_002823). Brain surfaces were reconstructed using recon-all (Dale et al., 1999; FreeSurfer 7.2.0, RRID:SCR_001847), and the brain mask estimated previously was refined with a custom variation of the method to reconcile ANTs-derived and FreeSurfer-derived segmentations of the cortical gray-matter of Mindboggle (Klein et al., 2017; RRID:SCR_002438).

#### 2.6.2 Functional data preprocessing

First, a reference volume and its skull-stripped version were generated using a custom methodology of fMRIPrep. Head-motion parameters with respect to the BOLD reference (transformation matrices, and six corresponding rotation and translation parameters) were estimated before any spatiotemporal filtering using mcflirt (Jenkinson et al., 2002; FSL 6.0.5.1:57b01774). BOLD runs were slice-time corrected to 0.911 s (0.5 of slice acquisition range 0 s-1.82 s) using 3dTshift from AFNI (Cox and Hyde 1997; RRID:SCR_005927). The BOLD time-series (including slice-timing correction) were resampled onto their original native space by applying the transforms to correct for head-motion. The BOLD reference was then co-registered to the T1w reference using bbregister (FreeSurfer) which implements boundary-based registration (Greve and Fischl, 2009). Co-registration was configured with six degrees of freedom. Several confounding time-series were calculated based on the preprocessed BOLD: framewise displacement (FD), DVARS, and two region-wise global signals. FD was computed using two formulations following Power et al. (absolute sum of relative motions; Power et al., 2014) and Jenkinson et al. (relative root mean square displacement between affines; Jenkinson et al., 2002). FD and DVARS were calculated for each functional run, both using their implementations in Nipype (following the definitions by Power et al., 2014). The two global signals were extracted within the CSF and the WM. The confound time series derived from head motion estimates and global signals were expanded with the inclusion of temporal derivatives and quadratic terms for each (Satterthwaite et al., 2013).

#### 2.6.3 Denoising

After preprocessing, further denoising was applied to the fMRI data, including high-pass filtering using a discrete cosine filter with 128 s cutoff, regression of volumes identified as motion outliers (frames that exceeded 0.5 mm FD or 1.5 standardized DVARS), and regression of the CSF and WM global signals and the six head motion parameters with their temporal derivatives and quadratic terms (4 CSF global signals, 4 WM global signals, and 24 head motion parameters in total; Satterthwaite et al., 2013).

### 2.7 Functional network construction

Automatic cortical parcellation was performed according to the Destrieux atlas (Destrieux et al., 2010) in the *FreeSurfer* software package (Fischl 2012), and subcortical segmentation was conducted using the FIRST algorithm (Patenaude et al., 2011) of the FSL toolbox (Smith et al., 2004). This resulted in a total of 164 regions of interest (ROIs) for each participant in their native (T1w) space. Functional connectivity matrices were constructed with the *Nilearn* toolbox (Abraham et al., 2014) by calculating pairwise Pearson correlations of detrended, standardized and denoised average time series of each ROI. Finally, Fisher transformation was applied for each correlation matrix.

### 2.8 Processing of diffusion weighted images and construction of the structural connectome

The acquisition and preprocessing of diffusion weighted images, tractography, and construction of structural networks are reported in detail elsewhere (Tolonen et al., 2023). Briefly, we collected 64 diffusion weighted (*b* = 1000 s/mm^2^) images, performed motion, eddy current, and susceptibility distortion correction (Andersson and Sotiropoulos, 2016; Irfanoglu et al., 2012), conducted constrained spherical deconvolution (Tournier et al., 2007) based anatomically-constrained tractography (Smith et al., 2012) from the white matter-gray matter interface, filtered the tractogram using SIFT2 (Smith et al., 2013, Smith et al., 2015), and constructed the structural connectome using the same ROIs as in the present study (Destrieux et al., 2010, Patenaude et al., 2011). All processing steps were performed with the FSL (Jenkinson et al., 2012) and MRtrix3 toolboxes (Tournier et al., 2019; RRID:SCR_024123).

### 2.9 Intervention procedure

After the pretest, the participants were assigned to either the experimental group or the active control group according to a lottery-based randomization (20 tickets for each group) conducted by an experimenter who was about to supervise the training period. The participants were unaware of the group to which they were assigned. Different experimenters supervised the pre- and posttest and training sessions, keeping also the experimenters conducting the assessments and the imaging sessions blinded to the participants’ group membership. During the training period, the participants trained three times a week, once in the laboratory (Institute of Behavioural Sciences, University of Helsinki) and twice at home, for five weeks. One training session lasted for approximately 25 min, and the participants were required to finish at least 10 training sessions to be invited to the posttest. A laptop computer was given to the participants to conduct the training sessions at home, but they were also allowed to use their own computer.

#### 2.9.1 Experimental group

The experimental group trained on a dual *n*-back task that was the same as in the pretest (see Section 2.4.1), except that each training session lasted for 20 blocks instead of 10. The program generated a logfile for each session that was stored on the computer. The participants also completed a questionnaire in which they reported the maximum *n*-level they achieved and their levels of motivation and arousal during the training session on a 10-point Likert scale (0 = very low and 10 = very high). Each participant who continued until posttest (see Section 2.10 Participant exclusion and study drop-outs) finished at least 11 training sessions, and 12 participants completed all 15 training sessions (mean number of sessions = 14.00, *SD* = 1.56).

#### 2.9.2 Active control group

The active control group played the Bejeweled II video game by PopCap Games from 2004. Their task was to score points by swapping jewels with adjacent ones, creating chains of same-colored jewels. This game was selected because it has low WM demands and it is broadly appealing, and because it has been successfully used in prior studies (e.g. Soveri et al., 2017b). The participants recorded their highest scores in a training log after each session. All participants who continued until posttest (see Section 2.10 Participant exclusion and study drop-outs) performed at least 11 training sessions, and 17 participants completed all 15 sessions (mean number of sessions = 14.65, *SD* = 0.99).

### 2.10 Participant exclusion and study drop-outs

Some participants were excluded from further analyses to ensure data quality. One NT participant was excluded due to unsuccessful cortical parcellation, resulting in 163 ROIs. Two ADHD participants were excluded from the resting state analyses because the imaging field during MRI was misplaced. Participants were also excluded from specific task analysis if their mean connectivity (mean of all pairwise correlations) exceeded 3 standard deviations from the group mean in the task. Furthermore, three ADHD participants (2 from the experimental group and 1 from the active control group) did not continue the training period to the posttest. The final group sizes were as follows: 41 participants with ADHD and 35 NT participants for the pretest visuospatial *n*-back analyses; 41 participants with ADHD and 34 NT participants for the pretest digit *n*-back analyses; 37 participants with ADHD and 33 NT participants for the pretest resting state analyses; 18 in the experimental and 19 in the active control group for the visuospatial *n*-back training effect analyses; 18 in the experimental and 20 in the active control group for the digit *n*-back training effect analyses; 17 in the experimental and 17 in the active control group for the resting state training effect analyses; and 19 in the experimental and 19 in the active control group for the structural connectivity training effect analyses.

### 2.11 Statistical analyses

#### 2.11.1 Normality inspections

The distributions of the scalar clinical and demographic variables were tested with the Shapiro-Wilk normality test and by visual inspection of the Q-Q plots. Variables determined to be normally distributed (age, Vocabulary, Matrix reasoning, mood, alcohol consumption, inattention, hyperactivity-impulsivity, and commission errors) were studied with independent samples *t*-tests when comparing groups or with Pearson’s correlation when studying associations between variables. Some scalar variables (age, Vocabulary, Matrix reasoning, and mood) were slightly curved resulting in rejection of normality in the Shapiro-Wilk test, and the group differences were double-checked with a nonparametric Mann-Whitney *U* test. The results remained the same. The omission error rates were not normally distributed and nonparametric tests (Mann-Whitney *U* test for group differences and Spearman’s *ρ* for associations between variables) were used for analyses. Group differences in gender, handedness, and education level were analyzed with the χ^2^ test.

#### 2.11.2 NBS

Network Based Statistic toolbox (Zalesky et al., 2010; RRID:SCR_002454) was used to search for functional networks differentiating ADHD and NT participants or associated with background variables, as well as to identify networks with group × session interactions in the WM training trial. NBS is a data-driven method that uses cluster-based thresholding for the correction of mass-univariate analyses. Briefly, subnetworks, or clusters, are first constructed from interconnected links (ROI-to-ROI connections) that exceed a primary threshold. Family-wise error (FWE) rate is then controlled by nonparametric permutation testing. The FWE-corrected *p*-level is estimated as the proportion of permutations in which the random-data cluster size (extent) or magnitude of the effect (intensity) is as large or larger than in the original data. Here, the group differences (pretest ADHD vs. NT) were studied with a one-sided *t*-test, associations with background variables (CPT error rates and ASRS scores) with Pearson’s correlations, and all interactions (task × group, group × WM load, intervention group × session, and intervention group × WM load × session) with repeated-measures ANOVA. We used primary thresholds of *t* = 3.0, 3.5 and 4.0, and their squares for *F*, and both extent and intensity as a measure of the cluster size. 10 000 permutations were performed in each test round. Statistical significance was set to *α* < 0.05.

### 2.12 Comparative reliability analyses

To ensure that our results are not bound to one denoising scheme (see Section 2.6.3 Denoising), we repeated all analyses with another denoising strategy. In this comparative analysis, movement outliers were not regressed out. Instead, participants whose movement exceeded 3 mm/radians for any of the six translational or rotational head movement parameters or whose mean FD exceeded 0.5 mm were excluded from the analyses. Otherwise, the same denoising options were used. All main effects remained similar, with small changes to the WM-related NBS networks. The results can be found in the Supplementary Results (Supplementary Figs. 1-2).

### 2.13 Brain network visualizations

The brain images were visualized with the *BrainNet Viewer* (Xia et al., 2013; RRID:SCR_009446).

## 3 Results

### 3.1 Behavioral characteristics

There were no statistically significant differences between the ADHD and NT groups in the general background variables (age, gender, handedness, general cognitive abilities, education level, mood, or alcohol consumption), but the ADHD group reported more inattention and hyperactivity symptoms than the NT controls and made more commission errors in the CPT. The proportion of correct responses during the single *n*-back tasks showed expected load effects, that is, lower hit rate with more demanding loads, but no group differences in hit rates were found in any load level. See Supplementary Tables 1, 2 and 3 in Supplementary Results for detailed characteristics regarding the sample used in the present study.

### 3.2 Group differences in functional connectivity

**Fig. 2:**
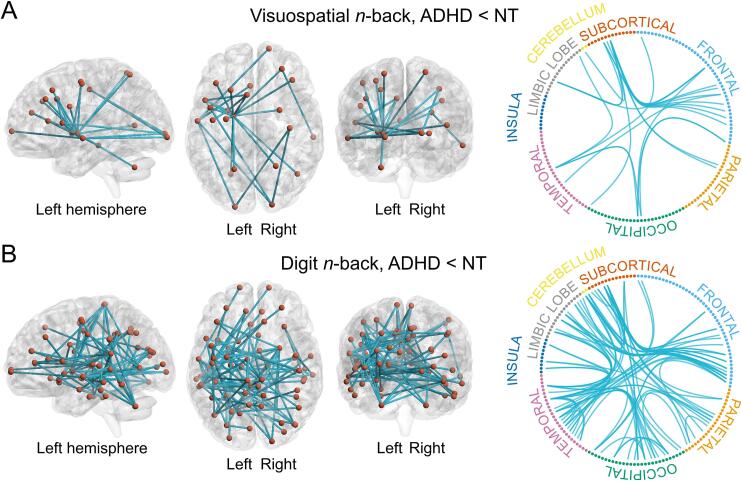
The brain networks with decreased functional connectivity in the ADHD group compared with the NT group during working memory tasks. A) The network related to the visuospatial *n*-back task included 25 edges and 23 nodes (primary threshold *t* = 3.5, *p* extent = 0.03, *p* intensity = 0.03, FWE-corrected). B) The network related to the digit *n*-back task included 91 edges and 73 nodes (primary threshold *t* = 3.5, *p* extent = 0.009, *p* intensity = 0.009, FWE-corrected). The networks were identical with extent and intensity as a measure of network size.

NBS identified a single network per WM task type (digit and visuospatial *n*-back) with lower functional connectivity in the ADHD group compared with the NT group (Fig. 2 & Supplementary Information). The networks were detected with both extent and intensity as a measure of network size and with all primary thresholds (*t*-values 3.0, 3.5, and 4.0), reducing in size with increasing thresholds (results with *t*-values 3.0 and 4.0 in Supplementary Fig. 3 in Supplementary Results). Both networks encompassed prefrontal areas, the cingulate, temporal, parietal and occipital cortices, cerebellum, and subcortical structures such as the dorsal striatum and globus pallidus. The digit *n*-back task-related network also included the insula, amygdala, and thalamus. The digit *n*-back task-related network included more edges than the visuospatial *n*-back task-related network. We found no WM-related networks with increased connectivity in the ADHD group compared with the NT adults (all *p*-values = 1.0), nor networks with significant task × group interactions (all *p*-values > 0.15), or statistically significant group × WM load interactions (all *p*-values > 0.12). There were no networks with significant group differences in the resting state condition (all *p*-values > 0.41).

### 3.3 Associations between functional connectivity and behavioral measures

**Fig. 3:**
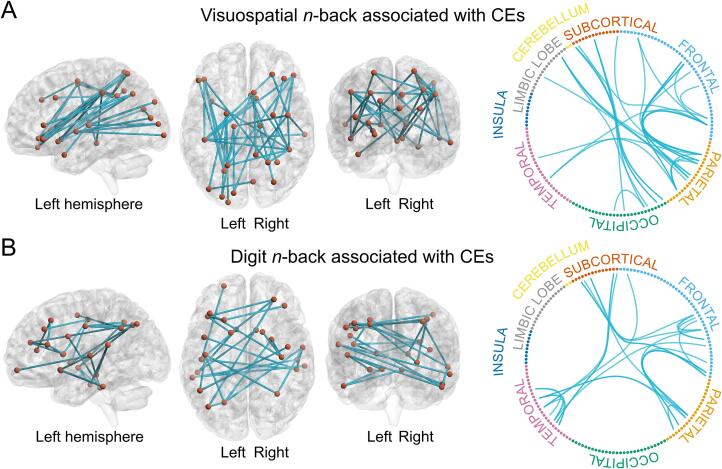
Networks in which WM-related functional connectivity was negatively associated with the number of commission errors (CEs) in the CPT. A) The network related to the visuospatial *n*-back task included 36 edges and 28 nodes (primary threshold *t* = 3.5, *p* extent = 0.02, *p* intensity = 0.02, FWE-corrected). B) The network related to the digit *n*-back task included 27 edges and 26 nodes (primary threshold *t* = 3.5, *p* extent = 0.03, *p* intensity = 0.03, FWE-corrected). The two networks were identical with extent and intensity as a measure of network size.

NBS revealed a single network per WM task type (digit and visuospatial *n*-back) in which functional connectivity was negatively correlated with the number of commission errors in the CPT (Fig. 3 & Supplementary Information). These two networks were detected with both extent and intensity as a measure of the network size. Both networks were identified with primary thresholds of *t* = 3.0 and *t* = 3.5, and the network related to the visuospatial task also with *t* = 4.0, reducing in size with increasing thresholds (results with *t*-values 3.0 and 4.0 in Supplementary Figure 4 in Supplementary Results). Both networks included frontal, temporal, and parietal areas, and the ventral and dorsal striatum. The visuospatial network included also occipital areas and the thalamus. The results remained highly similar even after correcting for performance (mean proportion of correct responses) in the *n*-back tasks. See Supplementary Table 3 for correlations between CPT and *n*-back performance and Supplementary Figure 5 for the connectivity results. There were no statistically significant WM-related networks identified with the other background variables (all *p*-values > 0.17). We found no networks with statistically significant associations with resting state connectivity (all *p*-values > 0.07).

### 3.4 Cognitive training intervention

We found no networks with a statistically significant intervention group × session interaction in either WM-related or resting state functional connectivity or structural connectivity with NBS (all *p*-values > 0.09). Also, the three-way interactions between intervention group, session, and WM load did not reach statistical significance (all *p*-values > 0.50).

In addition to whole-brain analyses, we conducted a post-hoc analysis examining the training effects on the networks differentiating the ADHD and NT groups. The training effects were analyzed using an intervention group × session design for each edge of the networks and the results were corrected for multiple comparisons with FDR correction. For functional connectivity, we chose edges with strongest group differences in the *t* = 3.0 networks (see Supplementary information) to restrict the analyses to the most relevant edges. We chose top 5%, top 10%, and top 20% of the edges in separate analyses to avoid possible bias caused by an arbitrary threshold. For structural connectivity, the whole *t* = 3.0 network was chosen for post-hoc analysis (see Supplementary Information; see Tolonen et al., 2023 for further group results) due to its smaller size. These analyses revealed no intervention group × session interactions surviving the correction of multiple analyses at any edge of the functional or structural networks. Some edges had significant interactions before multiple analyses correction. In the visuospatial *n*-back task, the experimental group showed more training-induced increases in functional connectivity compared with the active control group in connections from the opercular part of the left inferior frontal gyrus to the left pallidum (*F*(1, 35) = 8.14, *p* = 0.007), and from the left superior precentral sulcus to the left pallidum (*F*(1, 35) = 5.17, *p* = 0.029). In the digit *n*-back task, the active control group had more pronounced increase in functional connectivity after training than the experimental group between the left subcallosal gyrus and the left anterior occipital sulcus (*F*(1, 36) = 8.60, *p* = 0.006). The active control group also showed increased structural connectivity compared with the experimental group between right putamen and right superior occipital sulcus (*F*(1, 36) = 4.27, *p* = 0.046).

## 4 Discussion

In the present study, we examined alterations in functional connectivity during WM performance and resting state in adults with ADHD vs. NT controls and further investigated whether WM training intervention influences the functional or structural network configuration in the ADHD group. In accordance with our hypotheses, WM-related functional hypoconnectivity in adults with ADHD compared with NT adults was observed in widespread brain networks. Furthermore, as we hypothesized, we found that functional connectivity was associated with performance in an attention task regardless of diagnostic status. However, contrary to our hypothesis, no effects of WM training on functional connectivity were observed, and no intervention effects were detected in structural networks either. These findings shed light on the neural basis of aberrant WM functioning and its (limited) malleability to training in adult ADHD.

### 4.1 WM-related functional connectivity in adult ADHD: Group differences and associations with task performance

We observed decreased functional connectivity during visuospatial and verbal *n*-back tasks in adults with ADHD, which is in line with findings that individuals with this neurodevelopmental disorder have difficulties in both WM domains (Alderson et al., 2013). There were no significant differences in the connectivity patterns between task types. Furthermore, group differences in functional connectivity did not vary between separate WM loads, although it must be noted that the length of our fMRI data during each task load restricts the interpretation of the results (Van Dijk et al., 2010), which also prompted us to not analyse different load levels separately.

Hypoconnectivity during verbal WM task in adults with ADHD vs. NT controls was also reported by Wolf and colleagues (2009). In that study, hypoconnectivity was found in areas that appeared also in the networks of the present study, namely the ventral prefrontal cortex, anterior cingulate cortex, superior parietal lobule, and cerebellum, although they also reported hyperconnectivity in the prefrontal cortex, dorsal cingulate cortex, and cuneus. Decreased structural connectivity in overlapping networks was also observed in our previous study on adult ADHD (Tolonen et al., 2023). In most previous studies that have focused on children with ADHD, hyperconnectivity during WM performance has been a more common finding than hypoconnectivity (Bédard et al., 2014, Kowalczyk et al., 2023, Massat et al., 2012, Wu et al., 2017). However, WM performance evolves strongly across development (Gathercole et al., 2004), together with neural maturation of important WM hubs, such as the prefrontal cortex (Fuster 2001). Brain maturation also shapes connectivity patterns during WM performance (van den Bosch et al., 2014), as well as during other cognitive processes and rest (Stevens 2016), complicating comparisons between adult and child studies. Although direct evidence of age-related changes in WM-related aberrant activity in individuals with ADHD is still lacking, our results, together with the previous adult study (Wolf et al., 2009), suggest that maturation in this population can lead to the emergence of WM-related hypoconnectivity in adulthood. This might reflect the parallel tendency of the symptom manifestation shifting from overregulation (hyperactivity) to underregulation (inattention) from childhood to adulthood (Vos et al., 2022). Overall, our findings indicate that widespread aberrancies in functional networks underlie the challenges in verbal and visuospatial WM that individuals with ADHD commonly encounter.

The networks showing WM-related group differences included several brain areas previously associated with WM and ADHD. Brain areas regarded as core regions for WM, including the prefrontal cortex and parietal lobules (Wang et al., 2019, Yaple and Arsalidou, 2018, Yaple et al., 2019), figure regularly in findings of structural and functional abnormalities in ADHD (Cortese et al., 2012, McCarthy et al., 2014, Yu et al., 2023). Furthermore, fronto-striatal loops have been regarded as central both in ADHD pathophysiology and WM performance (O’Reilly and Frank, 2006, Cubillo et al., 2012), as have cerebro-cerebellar pathways (Salmi et al., 2010, Stoodley, 2012, Stoodley, 2016). Additionally, abnormal WM-related activation in the insula (Cortese et al., 2012) and structural alterations in the insula and cingulate cortex (Norman et al., 2016, Yu et al., 2023) in ADHD have also been found, with both areas linked to WM performance (Wang et al., 2019, Yaple and Arsalidou, 2018, Yaple et al., 2019). Finally, structural and functional differences in ADHD vs. NT participants have been reported in the pre- and postcentral gyri and many temporal and occipital areas (Norman et al., 2016, Yu et al., 2023).

In addition to examining differences between individuals with and without ADHD, we wanted to see whether variance in the capability to sustain attention or in symptom severity is associated with WM-related connectivity regardless of the participants’ diagnostic status. We found that the number of commission errors in the CPT, presumably reflecting impulsivity and lapses of sustained attention, was negatively associated with connectivity during both verbal and visuospatial WM. As the observed group differences, these findings are in accordance with our previous study, where higher rates of commission errors were associated with decreased structural connectivity (Tolonen et al., 2023). This link between sustained attention and WM-related connectivity may be related to tight coupling of attention and WM (Oberauer, 2019). The attention-related networks shared many brain areas with the ones showing group differences, and, overall, the effects cannot be fully separated due to the significant group difference in the measured error rate. Nevertheless, these results indicate that participants’ attention skills modulate how brain areas are synchronized during WM performance.

In contrast to the results related to WM, we did not find connectivity differences between groups or associations with background variables during resting state. Although resting-state connectivity is regularly studied in ADHD, with some meta-analytic evidence of disrupted default-mode network connectivity (Sutcubasi et al., 2020), convergence between individual studies appears to be limited (Cortese et al., 2021). Our findings of WM-related aberrant connectivity in ADHD, together with the lack of such differences in resting state, suggest that task-based settings can reveal important additional aspects of the connectivity divergences related to the cognitive domains with which individuals with ADHD struggle.

### 4.2 Training-related changes in functional and structural connectivity in adult ADHD

Although accumulating evidence from behavioral studies suggests that the effects of WM training are rather limited (Melby-Lervåg et al., 2016, Sala et al., 2019, Soveri et al., 2017a) and previous evidence of brain functioning malleability promoted by WM training in ADHD partially comes from studies that did not have proper control groups (Stevens et al., 2016), it was nevertheless unexpected that we found no effects of WM training on functional or structural brain connectivity. After all, regional training effects were reported in this same sample (Salmi et al., 2020) and there are studies in NT groups reporting modulations of brain connectivity as a response to WM training (Brooks et al., 2020, Constantinidis and Klingberg, 2016, Román et al., 2017, Zuber et al., 2021).

Several potential reasons could explain the discrepancy with previous findings of the malleability of brain connectivity in NT groups. Many of these studies (Jolles et al., 2013, Takeuchi et al., 2010, Takeuchi et al., 2013), including those using NBS (Román et al., 2017, Zuber et al., 2021), have not used a control group or have used only a passive control group. Employing an active control group has been observed to diminish cognitive training effects at the behavioral level (Cortese et al., 2015), and possibly the control group type might also have some effects at the neural level. Related to this, it is important that the control group has similar expectations about the training effects as the experimental group (Boot et al., 2013), as was the case in the present study (see Salmi et al., 2020). However, this has been rarely reported in previous cognitive training studies examining brain connectivity. In an earlier study, training effects were only observed at a specific WM load level, 2-back (Thompson et al., 2016). Also in the previous study reporting training effects in this sample (Salmi et al., 2020), modulations of brain activity were dependent both on the task load and the type of the *n*-back task (digit vs. spatial). However, we found no interactions between intervention group, pre/post-session and WM load level in the current study, indicating similar training effects on each WM load. Nevertheless, these results should be taken with caution, as the suboptimal imaging length per each load level can make them less robust (Van Dijk et al., 2010). Another factor that might affect the results is the lack of performance improvements after WM training in the single *n*-back tasks performed during fMRI. This could be partly due to situation-related factors like anxiety from the scanning process, which can interfere with task performance. The complexity of the underlying training effects, however, could only partially explain the lack of effects in the present study as the model in the present study was similar to that in the previous study. Regarding the NBS analysis, one could argue that the connectivity effects are too small to be revealed by whole-brain analysis, which requires rigorous correction for multiple comparisons. However, we did not observe any training-induced changes surviving correction for multiple analysis in the single edges either, even though they included connections relevant to WM, such as fronto-parietal (Wang et al., 2019, Yaple and Arsalidou, 2018, Yaple et al., 2019), fronto-striatal (O’Reilly and Frank, 2006), and cerebro-cerebellar (Salmi et al., 2010, Stoodley, 2012) loops. Training effects were observed in a few individual edges before adjusting for multiple comparisons, which could guide independent future research. However, the results might be somewhat arbitrary, given that some of the changes were accentuated in the control group. Yet another possibility is that the extent of WM training effects is negatively affected by initial problems with related cognitive processes (for rich-get-richer effects see, e.g., Foster et al., 2017, Jones and Berryhill, 2012, Wiemers et al., 2019). However, there are also studies reporting the opposite effects (Traut et al., 2021). All in all, our knowledge of training-induced malleability of brain functioning in ADHD remains rather limited, calling for continued research.

Recently it has become evident that, in general, cognitive training effects are limited mostly to the trained task and task settings that are close to the trained one (i.e., near transfer; Gobet and Sala, 2023). This was apparent also in the current sample where WM training gains were observed predominantly in the trained task and other WM tasks (Salmi et al., 2020). Thus, the benefits of cognitive training in ADHD are most probably primarily related to improved task performances in the cognitive domain that is being targeted, such as WM. Nevertheless, training multiple cognitive processes simultaneously has led to reduced inattention symptoms in meta-analyses (Cortese et al., 2015, Zou et al., 2024), suggesting that multiprocess training might be more efficient in rehabilitation (however, see Westwood et al., 2023). Also, examining the processes that lead to cognitive enhancement, such as the role of self-generated or examiner-instructed strategies (e.g., Fellman et al., 2020) could be a useful future direction. Given the limits of transfer, an even more beneficial approach could be to directly train tasks that are difficult for individuals with ADHD in real life (Corrigan et al., 2023; see also Merzon et al., 2022, Seesjärvi et al., 2023).

### 4.3 Limitations

It is noteworthy that the present sample does not fully represent the ADHD population, as we excluded participants with comorbid disorders that affect the majority of the population to avoid potential confounding factors. Individuals with no comorbid disorders can be considered to represent a subpopulation with fewer symptoms which potentially contribute to group differences in brain connectivity. Additionally, the careful matching of background factors between the ADHD and NT groups could diminish some of the group differences evident at the population level. Our sample was not large enough to examine how phenotypic variability, including the gender and age of the participants, which are known to contribute to symptom manifestation, may influence the findings. It is also possible that long-term use of stimulant medication contributes to the observed results in a way that cannot be disentangled due to the lack of treatment naive participants. Overall, ADHD is a highly heterogeneous disorder, and it would be optimal to acquire larger samples for neuroimaging studies to examine this variability. Furthermore, we did not observe differences in *n*-back task performance between the ADHD and NT groups. Although some previous studies have reported similar findings (Massat et al., 2012, Valera et al., 2005), this does have possible implications to the interpretation of differences in functional connectivity. Moreover, the chosen whole-brain analysis might conceal more subtle differences in smaller subnetworks, such as fronto-striatal loops, because of the need to correct for multiple analyses. This could in particular affect the WM training paradigm, because the effects of practice on functional or structural connectivity could be rather small and restricted to specific connections. The cluster-based correction approach is also unable to identify connectivity patterns that do not form a connected component if they are individually too small to survive multiple analyses correction.

### 4.4 Conclusions

The present study examined the neural underpinnings of WM deficits in adults with ADHD and tested whether aberrant connectivity can be modified via systematic training performed at WM capacity limits. As expected, functional connectivity was decreased in the adults with ADHD compared with NT adults in widespread brain networks. The group differences observed in visuospatial and digit WM tasks were partially overlapping. Unexpectedly, WM training that resulted in the modulation of regional activity (group × session interaction) in this same sample (Salmi et al., 2020), did not show up in the present functional connectivity analysis, and training had no effect on structural brain networks either. Altogether, the present study highlights that aberrancies in widespread networks underlie deficits encountered in ADHD, in agreement with the view of ADHD as a brain dysconnectivity syndrome but suggests that the possibility of ameliorating such network-level divergences by WM training can be limited. Future research is called for, hopefully with more effective WM training paradigms and larger samples which allow to account for the interindividual differences in training-related outcomes that may be particularly prominent in heterogeneous neurodevelopmental disorders such as ADHD.

## Author contributions

JS, ML, KA, PT, and SL designed the experiment. SL, AK, and PT selected, recruited, and diagnosed the patients. TT collected the data, processed the functional data, and performed all analyses. TR processed the structural data. JS and TT wrote the manuscript, which was commented, complemented, and agreed on by all authors.

## Ethics approval

The study was conducted in accordance with the Declaration of Helsinki. The study was reviewed and approved by the Ethics Committee for Gynecology and Obstetrics, Pediatrics and Psychiatry of the Helsinki and Uusimaa Hospital District.

## Consent to participate

All participants gave their written consent to participate in the study.

## Clinical trial registration

The clinical trial presented in the study was retrospectively registered on May 9, 2024, with a registration number ISRCTN42801302.

## CRediT authorship contribution statement

**Tuija Tolonen:** Writing – review & editing, Writing – original draft, Visualization, Investigation, Funding acquisition, Formal analysis, Conceptualization. **Sami Leppämäki:** Writing – review & editing, Resources, Investigation, Conceptualization. **Timo Roine:** Writing – review & editing, Funding acquisition, Formal analysis. **Kimmo Alho:** Writing – review & editing, Funding acquisition, Conceptualization. **Pekka Tani:** Writing – review & editing, Resources, Investigation, Conceptualization. **Anniina Koski:** Writing – review & editing, Resources, Investigation. **Matti Laine:** Writing – review & editing, Project administration, Funding acquisition, Conceptualization. **Juha Salmi:** Writing – review & editing, Writing – original draft, Supervision, Project administration, Funding acquisition, Data curation, Conceptualization.

## Funding

This study was funded by the Research Council of Finland (grant numbers for ML: 260276 & 323251; for KA: 260,054 & 297848; for JS: 325981 & 328954), Åbo Akademi University Endowment for the BrainTrain project (for ML), Finnish Cultural Foundation (for TR), and Vilho, Yrjö, and Kalle Väisälä Foundation of the Finnish Academy of Science and Letters (for TT). Open access funded by Helsinki University Library.

## Declaration of competing interest

The authors declare that they have no known competing financial interests or personal relationships that could have appeared to influence the work reported in this paper.

## Data Availability

The data that support the findings of this study are available on request from the corresponding author. The data are not publicly available due to privacy or ethical restrictions.
