# Supplementary material for: Working memory related functional connectivity in adult ADHD and its amenability to training: A randomized controlled trial
Source: Neuroimage Clin. 2024 Nov 2;44:103696. doi: 10.1016/j.nicl.2024.103696 (PMC11602582; doi:10.1016/j.nicl.2024.103696)
Supplement: Supplementary Data 1 [file mmc1.docx]

**Supplementary results**

**Article title**

Working memory related functional connectivity in adult ADHD and its amenability to training: a randomized controlled trial

**Authors**

Tuija Tolonen, Sami Leppämäki, Timo Roine, Kimmo Alho, Pekka Tani, Anniina Koski, Matti Laine, Juha Salmi

**Supplementary Tables** pp. 2–3

**Supplementary Figures** pp. 4–7

| **Supplementary Table 1.** *Demographic and clinical characteristics of the participants.* | | | | |
| --- | --- | --- | --- | --- |
| Characteristics | ADHD  (*n* = 42) | NT  (*n* = 35) | Test statistic (*df*) | *p* |
| Age, *M (SD)* | 28.67 (5.21) | 28.69 (7.76) | *t* (57.59) *=* 0.01 | .99 |
| Gender, male/female | 19/23 | 14/21 | χ² (1) = 0.21 | .64 |
| Handedness, right/left | 35/7 | 32/3 | χ² (1) = 1.11 | .29 |
| General cognitive abilities, *M (SD)* |  |  |  |  |
| Vocabulary | 11.21 (2.59) | 11.17 (2.44) | *t* (75) = -0.07 | .94 |
| Matrix reasoning | 12.29 (2.82) | 13.14 (2.25) | *t* (75) = 1.45 | .15 |
| Education* | 6/11/7/1/5/5 | 2/12/2/1/5/9 | χ² (5) = 5.74 | .33 |
| Mood, *M (SD)* | 5.02 (3.39) | 5.00 (3.73) | *t* (74) = -0.03 | .98 |
| Alcohol consumption, *M (SD)* | 4.26 (2.34) | 3.47 (1.97) | *t* (74) = -1.57 | .12 |
| ADHD symptoms (ASRS), *M (SD*) |  |  |  |  |
| Inattention | 23.25 (4.99) | 12.60 (5.51) | *t* (75) = -8.89 | < .001 |
| Hyperactivity-impulsivity | 19.33 (7.24) | 10.17 (5.03) | *t* (72.78) = -6.53 | < .001 |
| CPT performance (CPT-2) |  |  |  |  |
| Omission errors, *Mdn (Q1*–Q3*)* | 1 (0–3) | 1 (0–1) | *U* = 719 | .87 |
| Commission errors, *M* (*SD*) | 17.45 (7.02) | 10.06 (5.47) | *t* (75) = -5.01 | < .001 |
| Working memory performance, *M (SD*) |  |  |  |  |
| Digit span | 11.55 (3.05) | 12.06 (3.06) | *t* (75) = 0.73 | .47 |
| Visuospatial 1-back, hit rate (%) | 90.83 (11.70) | 92.46 (6.93) | *t* (74) = 0.72 | .47 |
| Visuospatial 2-back, hit rate (%) | 87.90 (13.58) | 85.37 (11.13) | *t* (74) = -0.88 | .38 |
| Visuospatial 3-back, hit rate (%) | 12.83 (10.89) | 10.57 (6.97) | *t* (74) = -1.06 | .30 |
| Digit 1-back, hit rate (%) | 92.78 (12.56) | 93.66 (5.03) | *t* (74) = 0.39 | .70 |
| Digit 2-back, hit rate (%) | 88.34 (13.32) | 86.17 (10.79) | *t* (74) = -0.77 | .44 |
| Digit 3-back, hit rate (%) | 15.07 (9.48) | 12.63 (6.94) | *t* (74) = -1.26 | .21 |
| *Note. df* = degrees of freedom, *M* = mean, *SD* = standard deviation, ASRS = Adult ADHD Self-Report Scale, CPT-2 = Conners Continuous Performance Test 2 *Mdn* = median, *Q1* = first quarter, *Q3* = third quarter. *Education scale in order of appearance: comprehensive school / upper secondary school / vocational school / community college level / bachelor’s degree / master’s degree. | | | | |

| **Supplementary Table 2.** *Correlations between self-reported ADHD symptoms and number of errors in the CPT. Associations with Omission errors with Spearman’s rho, associations between all other variables with Pearson correlation.* | | | | |
| --- | --- | --- | --- | --- |
|  | Inattention | Hyperactivity-impulsivity | Commission errors | Omission errors |
| ADHD (*N* = 42) |  |  |  |  |
| ADHD symptoms (ASRS) |  |  |  |  |
| Inattention | - |  |  |  |
| Hyperactivity-impulsivity | .53** | - |  |  |
| CPT performance (CPT-2) |  |  |  |  |
| Commission errors | .18 | .12 | - |  |
| Omission errors | .25 | .47** | .24 | - |
| NT (*N* = 35) |  |  |  |  |
| ADHD symptoms (ASRS) |  |  |  |  |
| Inattention | - |  |  |  |
| Hyperactivity-impulsivity | .53** | - |  |  |
| CPT performance (CPT-2) |  |  |  |  |
| Commission errors | .35* | .26 | - |  |
| Omission errors | .10 | .12 | .31 | - |
| *** p* < .01*, * p* < .05  *Note.* ASRS = Adult ADHD Self-Report Scale, CPT-2 = Conners Continuous Performance Test 2*.* | | | | |

| **Supplementary Table 3.** *Correlations between mean hit rates during the single n-back tasks and number of errors in the CPT. Associations with Omission errors with Spearman’s rho, associations between all other variables with Pearson correlation.* | | | | |
| --- | --- | --- | --- | --- |
|  | Visuospatial task hit rate | Digit task hit rate | Commission errors | Omission errors |
| *n*-back tasks |  |  |  |  |
| Visuospatial, hit rate | - |  |  |  |
| Digit, hit rate | .88** | - |  |  |
| CPT errors (CPT-2) |  |  |  |  |
| Commission errors | .03 | -.02 | - |  |
| Omission errors | -.19 | -.05 | .24* | - |
| *** p* < .01*, * p* < .05  *Note.* CPT-2 = Conners Continuous Performance Test 2*.* | | | | |


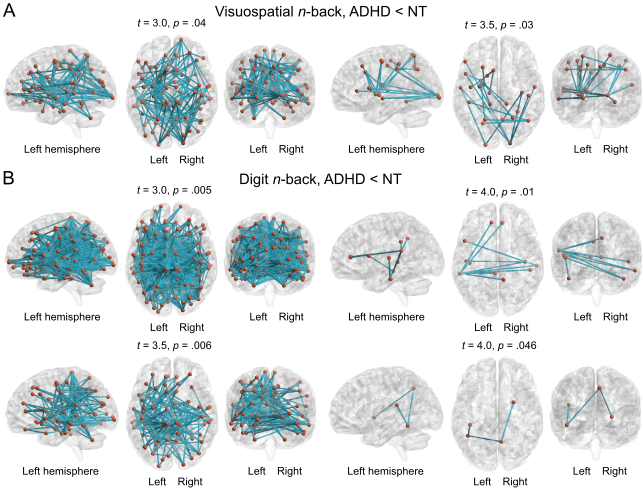


**Supplementary Figure 1.** Group results of the comparative analyses with a different denoising method in the fMRI data preprocessing. NBS identified decreased functional connectivity in the ADHD group compared with the NT group during working memory tasks. A) The network related to visuospatial n-back task included 128 edges and 81 nodes with t = 3.0 and 25 edges and 25 nodes with t = 3.5. No network was identified with t = 4.0. B) The network related to the digit n-back task included 534 edges and 138 nodes with t = 3.0 and 133 edges and 82 nodes with t = 3.5. With t = 4.0, two networks emerged, one with 10 edges and 11 nodes (upper), and one with 3 edge and 4 nodes (lower). All p-values are FWE-corrected.

**
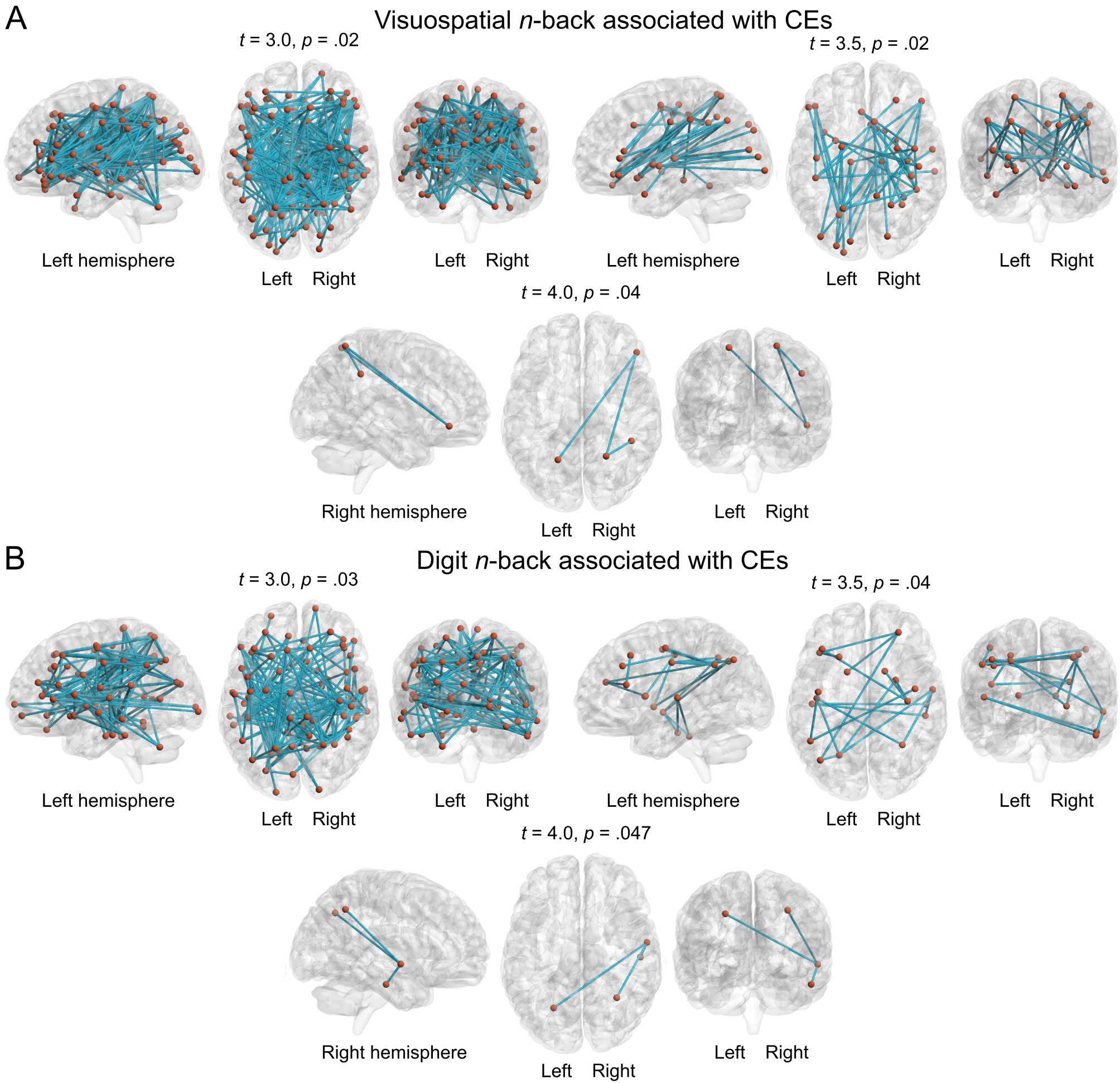
****Supplementary Figure 2.** Correlation results of the comparative analyses with a different denoising method in the fMRI data preprocessing. NBS identified networks in which WM-related functional connectivity was negatively associated with the number of commission errors (CEs) in the CPT. A) The network related to visuospatial n-back task included 231 edges and 102 nodes with t = 3.0, 42 edges and 32 nodes with t = 3.5, and 3 edges and 4 nodes with t = 4.0. B) The network related to the digit n-back task included 142 edges and 82 nodes with t = 3.0, 20 edges and 19 nodes with t = 3.5, and 3 edges and 4 nodes with t = 4.0. All p-values are FWE-corrected.


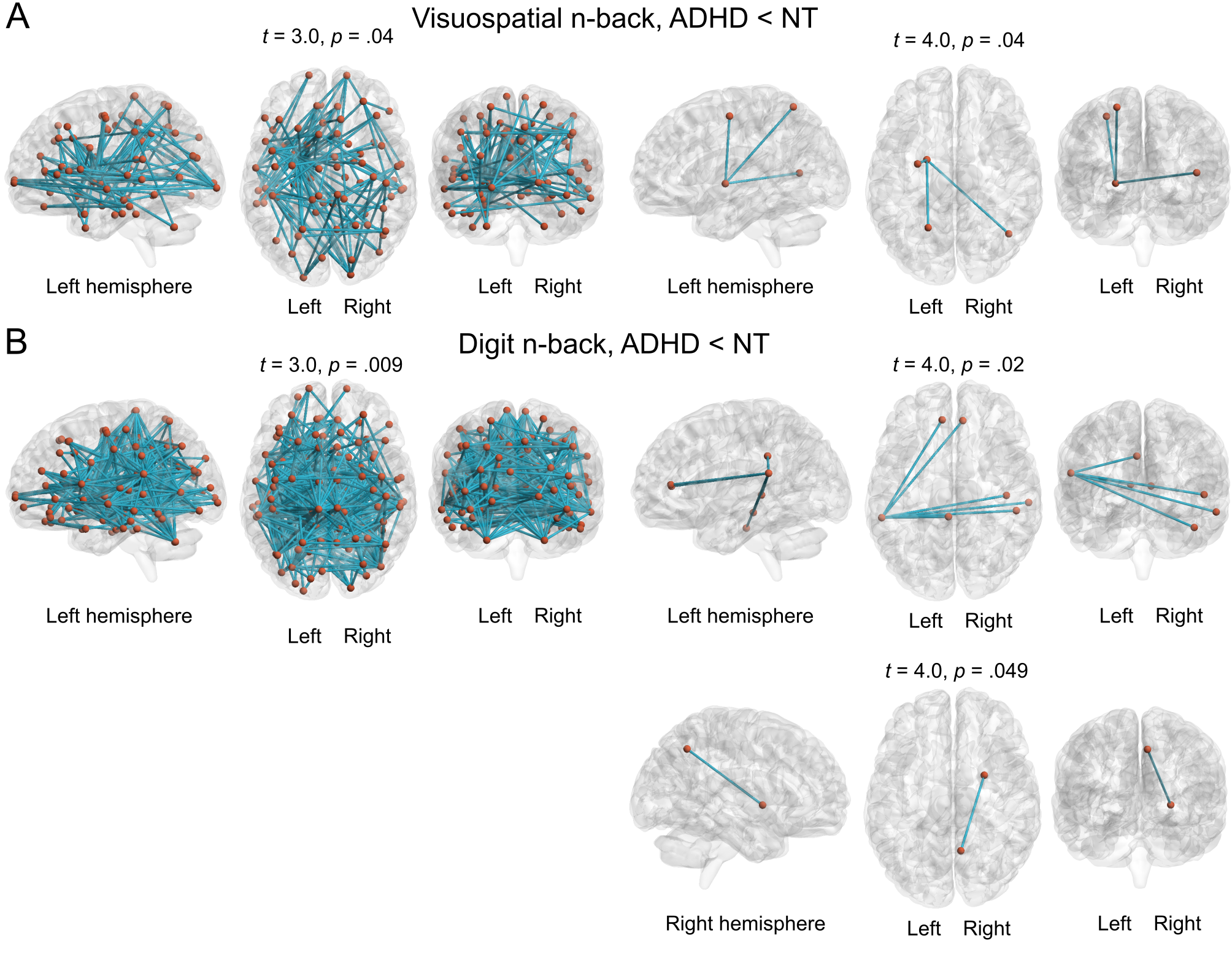


**Supplementary Figure 3.** The brain networks with decreased functional connectivity in the ADHD group compared with the NT group during working memory tasks with primary thresholds t = 3.0 and 4.0. A) The network related to visuospatial n-back task included 124 edges and 85 nodes with t = 3.0 and 3 edges and 4 nodes with t = 4.0. B) The network related to the digit n-back task included 383 edges and 131 nodes with t = 3.0. With t = 4.0, two networks emerged, one with 6 edges and 7 nodes (upper), and one with 1 edge and 2 nodes (lower). All p-values are FWE-corrected.

**
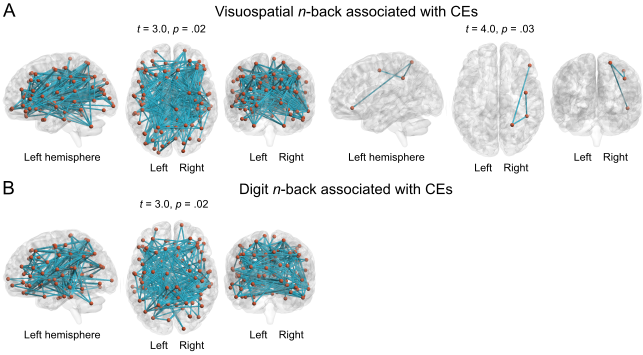
**

**Supplementary Figure 4.** The brain networks in which WM-related functional connectivity was negatively associated with the number of commission errors (CEs) in the CPT with primary thresholds t = 3.0 and 4.0. A) The network related to visuospatial n-back task included 250 edges and 107 nodes with t = 3.0 and 3 edges and 4 nodes with t = 4.0. B) The network related to the digit n-back task included 190 edges and 103 nodes with t = 3.0. No network was identified with t = 4.0. All p-values are FWE-corrected.

**
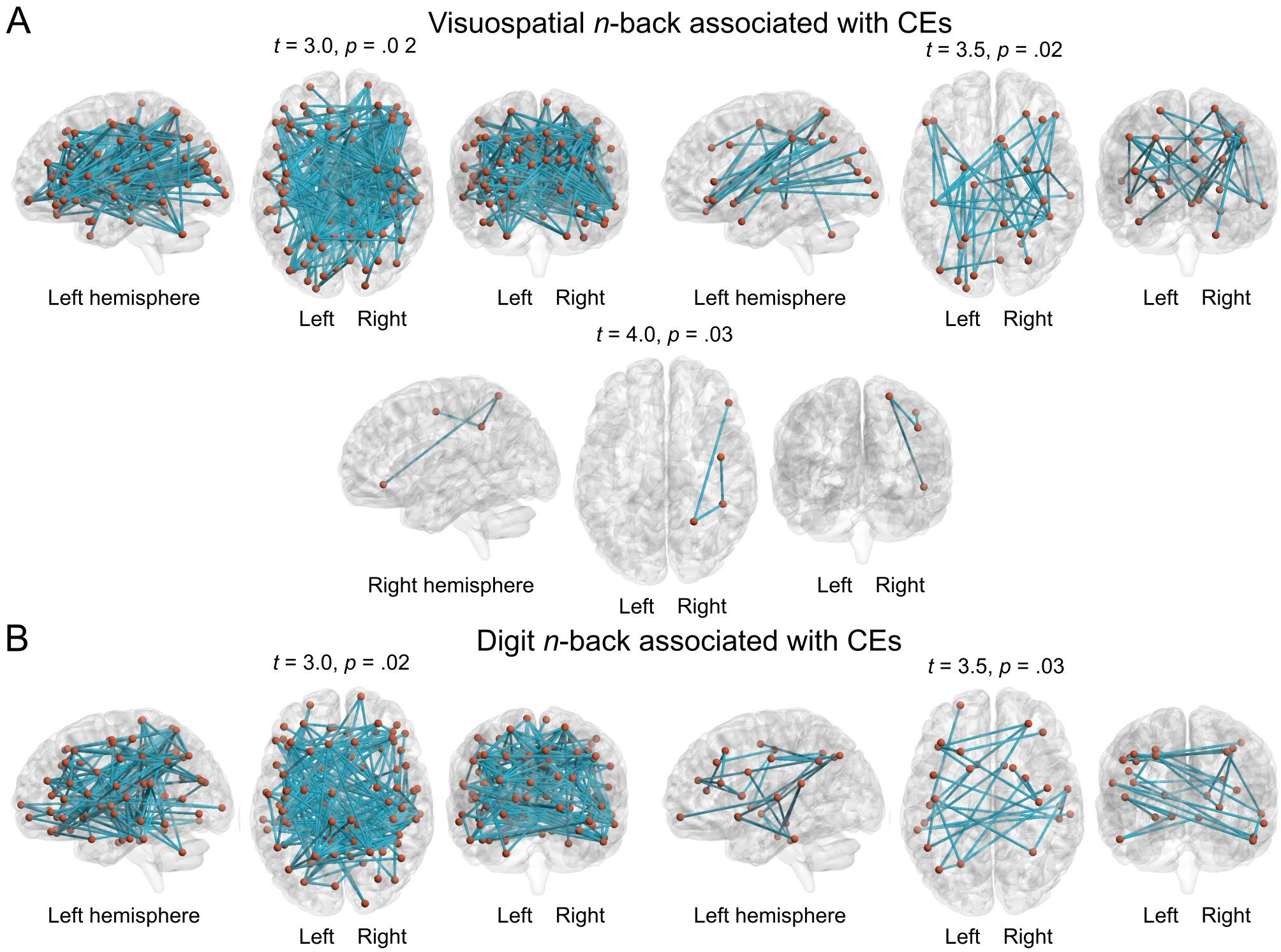
Supplementary Figure 5.** Correlation results of the comparative analyses controlling the participants’ performance during the single *n*-back tasks. NBS identified networks in which WM-related functional connectivity was negatively associated with the number of commission errors (CEs) in the CPT. A) The network related to visuospatial n-back task included 243 edges and 108 nodes with t = 3.0, 38 edges and 31 nodes with t = 3.5, and 3 edges and 4 nodes with t = 4.0. B) The network related to the digit n-back task included 185 edges and 101 nodes with t = 3.0, and 27 edges and 26 nodes with t = 3.5. No network was identified with t = 4.0. All p-values are FWE-corrected.
